# Singapore’s health reforms in response to an aging society

**DOI:** 10.1057/s41271-025-00592-8

**Published:** 2025-08-13

**Authors:** Divyansh Panesar

**Affiliations:** The University of Auckland, Private Bag 92019, Auckland, 1142 New Zealand

**Keywords:** Health policy, Health care reform, Singapore, Social values, Aged, Long-term care

## Abstract

Singapore’s shift toward a more collectivist model of healthcare marks a significant departure from its traditionally neoliberal approach, particularly in addressing the needs of its rapidly ageing population. The CareShield Life and Long-Term Care reforms aim to enhance financial protection, expand community-based services, and align health policy with evolving societal expectations. Nevertheless, questions remain about the adequacy of coverage, particularly in the context of rising chronic disease and long-term care costs. The move toward community care introduces important logistical and quality challenges. Singapore’s approach is unique in its extensive stakeholder engagement and values-based design. It reflects a pragmatic effort to reconcile long-standing principles of personal responsibility with growing demand for collective security in later life. Though outcomes will take time to manifest, the reform represents a notable case of policy realignment and offers transferable lessons for health systems navigating ageing and sustainability challenges.

## Key Messages

- Singapore’s shift toward collectivist long-term care financing reflects a new social contract: state protection without abandoning personal responsibility.
- Singapore’s CareShieldLife reform marks a significant shift from neoliberal principles toward collective long‑term care financing, demonstrating how even market‑oriented systems can evolve in the face of ageing demographics.
- These reforms show that stakeholder-driven, bottom-up policy design can succeed even in top-down systems.

## Introduction

Singapore currently ranks among countries with the highest global life expectancy, a status it aims to preserve through strategic public health management. As Singapore peers ahead, it must address rising demand for health services, particularly long-term care, and the financial pressures this entails. This raises the central empirical puzzle explored in this paper: why and how could a historically neoliberal health system adopt a collectivist long-term care policy?

### Political context

Historically, Singapore’s health and social policies have been rooted in a neoliberal, productivist ethos that emphasises individual responsibility, family support, and limited state-administered welfare provision [1, 2]. Like many post-colonial republics, Singapore embraced self-sovereignty and advanced through socio-economic and structural developments with neoliberal ideology [2]. However, unlike many of its contemporaries that sought to dismantle colonial legacies, Singapore leveraged selected colonial institutions to compete effectively on the geopolitical front [2]. This selective continuity, rather than rupture, enabled exceptional growth while consolidating technocratic state control.

Singapore initially redistributed wealth through asset enhancement rather than targeted subsidies, avoiding excessive budgetary burdens and delivering rapid economic growth [3]. At the time Singapore boasted a youthful demographic and the majority of Singapore’s population constituted the working class, with only 2.5% of the mid-twentieth century population being aged above 65 [4]. This allowed for a competitive industrial advantage on the global front, with Singapore evolving into the modern capitalist powerhouse of today [4].

Consistent with its productivist orientation, social and health policies prioritised fiscal prudence over collectivist-welfare principles. Singapore's health was shaped heavily by its political context where state support was provided only after individual contribution [5, 6]. This ideology prioritised cost containment and personal responsibility over redistributive goals, limiting overutilisation or wastage of health resource [6]. While such policies drew criticism for their limited focus on health equity, the initial World Health Organisation report on health systems performance demonstrated that Singapore ranked high [5, 7]. Indeed, it may be argued that Singapore’s healthcare system historically outperformed most of its global counterparts [8].

These early successes reinforced Singapore’s departure from state-led service delivery, positioning the state not as a sovereign provider, but as a regulator and supervisor of market provision. [9]. Health was framed primarily as an individual rather than societal responsibility with the development of personal savings (Medisave) and social insurance (Medifund) schemes [10]. Like other industrial nations with a young, employable population, the ideals of self-reliance were within societal interest [9]. The policies were economically robust and allowed Singaporeans to remain relatively healthy during a period of rapid development [11].

However, as Singapore advanced, so too did the age of its population at an unprecedented rate [12]. In the mid-2010s, rising healthcare costs and widening coverage gaps became politically salient, prompting policymakers to expand risk-pooling mechanisms and reframe collective responsibility as moral or social imperatives [13]. The idea that personal responsibility alone could sustain health financing became increasingly untenable, especially with an aging population unable to participate in the workforce, the very condition upon which prior entitlements were premised [14]. Calls for more comprehensive and universal health provisions grew, with affordability emerging as a central concern.

### Health system context

The last twenty years have seen Singapore progress from aging, to aged, and now requiring a new moniker; a ‘super-aged’ society [15]. By 2025, more than 20% of the population is projected to be aged 65 or older, presenting the challenge of reconciling personal liberty with the societal obligation to provide for an increasingly frail elderly population [11]. Early projections revealed that a majority of older Singaporeans lacked sufficient MediSave savings to meet the costs associated with later life, particularly those related to long-term care (LTC) [16]. The existing system became increasingly ill-equipped to finance or deliver care for chronic disease and functional decline—both more prevalent with age [17]. The initial government strategy, relying on legal and moral appeals to filial piety via the Maintenance of Parents Act, was met with heavy criticism [18]. While the policy aimed to reinforce traditional caregiving norms, critics argued that it ignored economic realities and risked generating intergenerational conflict [18]. Once again, shifts in public opinion catalysed social policy change, prompting a turn toward more collective approaches to healthcare [19].

Although there existed a welfare scheme for elderly (Eldershield), it required purchasing through private insurers thus limiting its access [14]. While coverage was nominally wide, the benefits were shallow and modest [12]. In fact, many Singaporeans were unaware of the program and unsure of either involvement or coverage [20]. Eldershield was subsequently revealed to provide insufficient remuneration for health care with benefits that remained unadjusted for inflation, leading households into financial anxiety [19]. The monthly cash benefit of S$400 fell short of the minimum salary required for foreign domestic helpers, who are often employed in Singaporean households to care for the elderly [19].

Previously exemplary by many standards, Singapore began showing shortfalls in the way it addressed care for its elderly [21]. These gaps became evident when examined through WHO’s health system objectives (*responsiveness to population expectations, betterment of population health, and protection against the financial burden of ill health*) [7]. National surveys and health user consultations revealed that population expectations were not being met; especially with regards to financial protection against cost of ill health [12]. Age-stratified expenditure data further showed that those aged 40 and above accounted for a disproportionate share of national healthcare spending [22]. These pressures provided a powerful impetus for reform. Public opinion of health priorities now included ensuring affordability, especially for its elderly [23]. In response, the government studied similar cases across the world and began development of its own reforms, eventually releasing the *CareShield Life and Long-Term Care Bill*, 2019 (CSL-LTC bill).

### The empirical puzzle

In analytical terms, an empirical puzzle emerges: how does a market-oriented, libertarian-inclined state like Singapore come to adopt a collectivist long-term care model? Singapore’s embrace of a mandatory public LTC insurance scheme stands in contrast to its historical reputation for minimal welfare and resistance to redistributive social insurance. This pivot towards greater collectivism suggests a recalibration of Singapore’s social contract in response to emerging challenges. It reflects a recognition that a rapidly aging population and rising care needs may necessitate stronger social safety nets than the traditional neoliberal model can provide – and may exceed the capacities of individual and family-based financing [19].

The adoption of CareShield Life raises important questions about political feasibility. Its success reflects the confluence of multiple pressures: demographic trends, rising care costs, and evolving public expectations; that together generated the impetus and sociopolitical receptivity to reform. Just as crucial was its astute policy design: by framing CareShield Life as a self-funded insurance scheme with state-administered subsidies, rather than a tax-financed entitlement, the government preserved ideological continuity with its emphasis on personal responsibility while still substantially increasing risk-pooling [24]. In this way, Singapore has reconciled its market-driven instincts with a collectivist solution carefully tailored to fit local values. The result offers a valuable case study of the conditions under which even a resolutely market-oriented state can expand its social policy toolkit in response to structural shifts.

## Reform Implementation

### Long term care

CareShield Life (CSL), replaced the ElderShield program and brought care for older people to the forefront of Singapore’s health policy agenda [16]. Rather than a reactive response to public pressure, CSL marked an alignment with ongoing shifts in the nation’s healthcare ideology and a pragmatic adaptation to emerging social support needs. Instead of an optional insurance scheme that selected against those with comorbidities, CareShield Life is a compulsory scheme for those over 30 regardless of pre-existing disability [25]. The scheme retains fixed monthly cash benefits but removes the six-year cap on payout duration and ensures that benefit amounts increase over time [12, 26]. Drawing on international examples, the policy embeds indexation to reflect inflation and escalating care needs, while also removing restrictions on the type of care covered [26, 27]. This enables beneficiaries to pursue community-based and holistic care pathways, rather than being channelled into institutional or prescriptive models of support.

This reform directly addressed longstanding criticisms of preceding LTC models whilst retaining the co-payment strategies that had previously served well. Recognising that elderly individuals might be disadvantaged by employment history, disability, or lack of savings, the government introduced ElderFund the following year a supplementary scheme to support out-of-pocket costs such as insurance premiums for those unable to afford them [22].

The implementation of the CSL-LTC act was notably judicious, beginning with stakeholders engagement from ground up [20]. This approach allowed values-based policy to develop in line with public opinion, liberating government from manufacturing social momentum and enabling its Ministry of Health to focus on program-building and operational logistics [28]. The staggered rollout, initially targeting younger cohorts, allowed policymakers to monitor enrolment, premium collection, claims experience, and public reception, creating space for refinement prior to wider expansion. Importantly, the COVID-19 pandemic served as momentum for enacting healthy public policy, avoiding stresses on the adaptive capacity of the system [16, 29].

### Policy administration

Given the requirement for continuous, long-term efforts, leadership for policy implementation has been secular with a multi-faceted approach [28]. Policy enactment is coordinated by four authorities under the stewardship of the Ministry of Health: Ageing Planning Office (APO), Agency for Integrated Care (AIC), Regional Health Systems, and MoH Holdings [28]. These agencies collectively oversee the restructuring of the LTC sector to improve accessibility, quality, and affordability for an aging population. Among them, the Ageing Planning Office plays a particularly direct role in shaping and implementing ageing-related initiatives, with a strong focus on public engagement and promoting healthy longevity [30]. The Ministry of Health retains oversight of the overall health and LTC systems, including strategic policy direction, demand forecasting, financing mechanisms, regulatory governance, and inter-agency coordination [28].

Within this structure, the Ageing Planning Office serves as principle custodian of LTC strategy and ensures alignment with broader frameworks, including the CareShield Life (CSL) scheme. Notably, however, CSL is governed independently by the CSL Council, appointed in 2020, which oversees the actuarial sustainability, benefit adjustments, and long-term risk management of the scheme [27]. This division of governance reflects a model of distributed leadership that enables adaptability while maintaining policy coherence.

A major outcome of this administrative arrangement has been the Action Plan for Successful Ageing, a S$3 billion national blueprint developed following a year-long society-wide consultation process [31]. Its second iteration co-opted the private sector to update and ensure the relevance of the initiatives for current and future cohort of seniors [31]. This co-production model reflects Singapore’s broader shift from top-down technocracy toward more participatory and pluralistic governance in health and ageing policy. In parallel to these shifting paradigms in Singapore's health governance, the LTC strategy has further developed the ‘Healthier SG (Singapore)’ initiative as proactivity preventing illness [32]. The plan seeks to expand healthcare capacity across primary, acute, and LTC sectors, and is supported by investments in workforce training and system-wide care integration [32].

Ultimately, the reform of aged care in Singapore represents a paradigm-level intervention, redefining not only instruments and actors, but the underlying goals and assumptions of the system [33]. Such reforms are complex, politically ambitious, often non-linear in their evolution, with trajectories that are difficult to predict [34]. Furthermore, such reforms require developments not only in health systems, but also across lateral industries such as education, transport, and housing [16]. While early progress has shown promise, future iterations of Singapore’s LTC strategy will be critical tests of its institutional resilience and policy adaptability.

## Reform Implications

### Responding to public expectations

In some respects, Singapore’s current reforms contradict twentieth century polities of economic neoliberalism and, in theory, could risk poor performance across WHO’s parameter of responding to people's expectations. Accordingly, an extensive qualitative analysis of over 800 citizens, stakeholders, and experts, was performed through a range of conferencing [20]. While many participants did not explicitly contemplate their own future need for LTC, there was widespread dissatisfaction with the existing status-quo [20]. Interestingly, participants approved increased premiums and greater state involvement, a view well in contrast to twentieth century developmentalism [35]. It is likely that the values of filial responsibility allowed this very socialist opinion to survive alongside the pre-existing capitalist axiom [21]. Additionally, a broader narrative shift appears to be underway with younger voters and the expanding elderly demographic voicing growing concern over healthcare costs, inadequate coverage, and inequities in service access [36].

Singaporean households indicate a preference for community-based care over institutional or hospital-based models [20]. This is in keeping with global public opinion, although Singapore is the first to conduct such extensive stakeholder engagement for policy validation [37]. The CSL reform can therefore be seen as a response to this changing public opinion. Furthermore, global health models suggest integrated community health services are associated with improved elder health [38]. As a result, the LTC reform strongly supports the expansion of community-delivered services, including Senior Care Centres and Home Nursing, both of which address core health and social needs of ageing citizens [21].

In this light, principles of personal responsibility may coexist with those of community values, although there is the challenge of providing consumer autonomy despite mandatory participation. Care needs are often variable between households, with many services provided informally by families or caregivers [26]. Accordingly, various interventions are funded through CSL and its regular cash payments provide a degree of freedom when choosing between arrangements [39]. The scheme appropriately recognises diverse eligibility pathways and varying care needs, acknowledging that aged care in Singapore is often delivered in hybridised arrangements involving family, community, and institutional actors [28, 40].

Theoretically, Singapore’s case suggests that even authoritarian-capitalist states may expand social protection when facing demographic shifts and rising middle-class demands—a pattern distinctly noted in East Asian welfare capitalism [35]. At the same time it also challenges the notion that neoliberal frameworks are inherently intransigent; rather, Singapore demonstrates that under certain conditions, a shift toward collectivist policy can occur without upending the existing political order.

### Financial protection

Singapore’s health system has historically relied on substantial out-of-pocket (OOP) payments for insurance premiums, primary care, and lower-cost health services [12]. Criticisms of this structure, whether misunderstood or appropriately evaluated, are perhaps why Singapore ranked comparatively low in the WHO’s early financial-protection metrics [41]. Moreover, Singapore was a comparatively late adopter of reforms aimed at strengthening its primary care sector, an area critical to reducing costs in the aged care contex [16].

The CSL-LTC bill is represents one of Singapore’s most direct responses to the financial burden of disease in recent decades, as many of its initiatives target affordability and remuneration [27]. The government’s promotion of CSL has explicitly emphasised its role in mitigating the financial risks associated with long-term care [39]. Of the six state-advertised benefits of CSL, only one (lifetime coverage) is unrelated to direct financial implications.

The reform also addresses the potential impact of economic inflation and commits to increasing monetary benefits over time via indexed benefit increases [20]. Complementary to CSL, Healthier SG offers further incentives for preventive care, such as full subsidies for national screenings and vaccinations, waived co-payments for chronic care management under MediSave, and introducing a new drug subsidy tier under the Community Health Assist Scheme [32]. Behavioural incentive programmes, such as the National Steps Challenge offer rewards for enrolling—further reinforcing fiscal and personal health benefits.

Significant out-of-pocket fees are still necessary however, as the average cost of long-term care in Singapore is approximately four times higher than the regular payments provided by CSL [19]. While the CSL–LTC reforms are ideologically novel in Singapore’s context, they still assume that families or individual savings will finance the bulk of elder care. Austerity measures introduced in the last two decades have curbed some of this expenditure, outside of aged care, nearly one-third of overall health expenditure remains privately financed through out-of-pocket payments [42].

It is perhaps too early to definitively assess the monetary implications of the CSL-LTC reform, however, preliminary evaluations suggest that the level of financial protection offered remains modest. While there is an assurance that no Singaporean will be left entirely unsupported in the event of severe disability, there is no guarantee of full coverage for long-term care needs. Nonetheless, the reform establishes a significant policy shift as financial protection is now a central focus of system design, with mechanisms in place for further fiscal expansion in future iterations.

### Improving public health

In the pre-COVID era, Singapore achieved remarkable disability and health-adjusted life expectancy outcomes despite modest expenditure [43]. Using conventional metrics, the country presents as a healthy nation with a low burden of disease [2]. However updated recommendations from the Organisation for Economic Co-operation and Development urge a shift from siloed health provision integrated care models [44]. This approach highlights alignment across system hierarchies and cooperation across sectors, disciplines, and industries involved in social welfare and health. Evaluating the impact of the CareShield Life and Long-Term Care (CSL–LTC) Bill remains premature, as subsidiary legislation was only finalised in November 2021 [45]. The policy’s outcomes will likely require a longer timeframe for meaningful assessment.

Viewed through the lens of integrated care, however, efforts to improve population health reveal potential gaps. For example, although many older adults do not meet diagnostic thresholds for dementia or neurodegenerative disease, nearly 11% have are considered cognitively frail—a condition associated with adverse outcomes including functional decline, reduced quality of life, and increased mortality [46]. While Singapore has made progress on nutrition, physical activity, and obesity prevention for younger populations, similar metrics for older adults are less explored [16]. Integrated care plays a crucial role in facilitating the early identification of frailty, personalised health plans, family involvement, and monitoring or adjusting care plans [47, 48]. By addressing the medical and social aspects of health, such integrations can reduce hospitalisations and improve wellbeing.

While the CSL–LTC Bill does not explicitly reference integrated care in its legislative texts, its implementation methodology involves greater intersectoral data recognition and cross-industry alignment of health metrics [21]. More directly, the Healthier SG strategy works with community partners such as the Agency for Integrated Care to strengthen primary care and provide interdisciplinary approaches for care coordination [32]. Collaborative efforts between family doctors, care teams, and healthcare clusters are expected to provide well-synchronised, consistent care, supported by standardised protocols and interconnected information systems. Scaling up community-based care to meet the burgeoning demand, however, is a formidable undertaking and constitute a critical arena where Singapore’s LTC reform will be tested. Success will require sustained investment in the healthcare workforce, continuous improvement, and careful calibration of incentives to favour home and community-based solutions.

## Conclusions

Recent reforms are a noteworthy shift in Singapore’s healthcare approach, from a historically individualistic model to one increasingly shaped by collectivist principles. These strategies are bold, balancing Singapore’s foundational ethos of personal responsibility and embracing collective risk-sharing. With the world’s eyes watching, Singapore’s journey towards inclusive and sustainable healthcare models may serve as blueprint for those grappling with similar challenges. First, its strategy underscores the value of early, anticipatory action: implementing long-term care financing mechanisms before the full weight of population aging materialises. Second, stakeholder engagement and bottom-up input have helped build legitimacy and consensus, building public trust and making reform more politically sustainable. Third, the reforms demonstrate how mandatory but subsidised social insurance can expand coverage while preserving fiscal sustainability and personal responsibility. Fourth, institutional capacity and political continuity are critical enablers of reform and finally, reform must also proceed incrementally, using a phased approach that allows for adaptation and sustained political support. While Singapore's model offers valuable insights, it is important to consider relative differences in cultural, social, and economic factors that might impact the applicability of these reforms in different countries. Its model may be context-specific; however, the core principles of gradualism, risk-pooling, and stakeholder consensus offer a transferable framework for the realities of aging societies.
